# Exploration of D0_22_-Type Al_3_TM(TM = Sc, Ti, V, Zr, Nb, Hf, Ta): Elastic Anisotropy, Electronic Structures, Work Function and Experimental Design

**DOI:** 10.3390/ma14092206

**Published:** 2021-04-25

**Authors:** Guowei Zhang, Fenger Sun, Heping Liu, Xiaoyan Ren, Hong Xu, Mingjie Wang, Yizheng Fu

**Affiliations:** 1 School of Material Science and Engineering, North University of China, Taiyuan 030051, China; peace666@126.com (H.L.); xuhong@nuc.edu.cn (H.X.); 15513882577@163.com (M.W.); fuyizheng@nuc.edu.cn (Y.F.); 2 Department of Mechanical Engineering, Taiyuan Institute of Technology, Taiyuan 030008, China; renxiaoyan03@126.com

**Keywords:** trialuminides, structural properties, mechanical anisotropy, work function, experimental design, orientation relationship

## Abstract

The structural properties, elastic anisotropy, electronic structures and work function of D0_22_-type Al_3_TM (TM = Sc, Ti, V, Y, Zr, Nb, La, Hf, Ta) are studied using the first-principles calculations. The results indicate that the obtained formation enthalpy and cohesive energy of these compounds are in accordance with the other calculated values. It is found that the Al_3_Zr is the most thermodynamic stable compound. The mechanical property indexes, such as elastic constants, bulk modulus, shear modulus, Young’s modulus, Poisson’s ratio, and Vickers hardness are systematically explored. Moreover, the calculated universal anisotropic index, percent anisotropy and shear anisotropic factors of D0_22_-type Al_3_TM are analyzed carefully. It demonstrates that the shear modulus anisotropy of Al_3_La is the strongest, while that of Al_3_Ta is the weakest. In particular, the density of states at Fermi level is not zero, suggesting that these phases have metal properties and electrical conductivity. More importantly, the mechanisms of correlation between hardness and Young’s modulus are further explained by the work function. Finally, the experimental design proves that D0_22_-Al_3_Ta has an excellent strengthening effect.

## 1. Introduction

With the increasing demand of aerospace and automotive industry for structural material properties, aluminum rich compounds containing transition metal (TM, i.e., Sc, Ti, V, Y, Zr, Nb, La, Hf, Ta) elements have attracted extensive attention [1,2,3]. Among them, trialuminides (Al_3_TM) are the most potential candidate compounds, mainly because it can meet a variety of advantages, such as a high melting point, good thermal conductivity, low temperature damage resistance, strong creep resistance, and high specific strength. Furthermore, most of these intermetallics have different crystal structures of L1_2_, D0_19_, D0_22_, or D0_23_ [4,5,6,7,8,9,10,11]. Usually, the Al_3_TM series of trialuminides can crystallize into the cubic L1_2_ (space group Pm$\bar{3}$m) and tetragonal D0_22_ (space group I4/mmm) crystal structures. The L1_2_ structure has better ductility due to its higher symmetry and more slip systems. However, it is generally believed that the lower symmetry of D0_22_ structure is the main cause of poor ductility. Many attempts had been made to convert D0_22_ into L1_2_, which can make the aluminide have good ductility [12,13,14,15]. However, the D0_22_ structure has a good strengthening effect in the recent design of high entropy alloy. Hereinto, one of the critical challenges is to explore the internal mechanism of D0_22_-type trialuminides.

Previous studies have made a lot of efforts to reveal the properties of these intermetallic compounds. Recently, Jahnatek M et al. [16] investigated the interatomic bonds and the tensile anisotropy of Al_3_(Sc, Ti, V, Cr) by using density-functional theory. Here, in both the L1_2_ and D0_22_ crystal structure, the main bonding character originates from the saturation of dominant d^3^ (L1_2_) and d^4^ (D0_22_) hybrid orbitals located on the TM atoms. In addition, the structural, electronic, and thermodynamic properties of Al_3_(Ti_x_V_1−x_) alloy in D0_22_ and L1_2_ structures have been reported using the full-potential linearized augmented plane wave (FP-LAPW) method within the framework of the density functional theory (DFT) [17]. It can be drawn from this result that D0_22_ is the relatively stable phase used in these materials, while L1_2_ is always the metastable phase. In addition, the phase of Al_3_(Ti, V) crystal and electronic structure in D0_22_ and L1_2_ were studied in detail, which indicates that the increase of charge density along the Al-(V, Ti) bond is a characteristic of bonding [18,19]. Meanwhile, Schwarz et al. [20] had explored the properties of trialuminides with ultra-fine microstructures. It can be concluded that the D0_22_ structure has poor ductility because of low symmetry, which may be related to the insufficient number of slip systems in polycrystals. Chen Z et al. [21] had investigated the thermodynamic, elastic, and electronic properties of D0_22_-type Al_3_V and Al_3_Nb intermetallics under pressures using the first-principle method. Under the same pressure condition, the relative volume change of Al_3_Nb is smaller than that of Al_3_V, which is mainly because the bulk modulus of Al_3_Nb is larger than that of Al_3_V. By employing the Vienna ab initio simulation package (VASP), the relative stabilities of L1_2_, D0_22_ and D0_23_ with different structures in the intermetallic compound ZrAl_3_ were studied by Colinet C et al. [22]. The last theoretical calculations had shown that the structure of D0_22_ is slightly more stable than that of L1_2_. Li R et al. [23] studied the structural stability, electronic structures, and thermodynamic properties of HfAl_3_ with L1_2_, D0_22_ and D0_23_ different structures by the first-principle method. The calculated results revealed that the order of their structural stability can be arranged as D0_23_>D0_22_>L1_2_. Furthermore, Boulechfar R et al. [24] conducted the investigation of the structural stability, electronic, and thermodynamic properties of Al_3_Ta intermetallic compound by using the full-potential linearized augmented plane wave (FP-LAPW) computational approach. The conclusion is that the total energy and the total density of states at the Fermi level indicate that the stability of D0_22_ structure is better than that of D0_23_ and L1_2_ structures. Li C et al. [25] calculated the properties of intermetallic compound Al_3_TM (TM = Ti, Zr, Hf, Sc) and the interfacial properties of Al_3_TM (TM = Ti, Zr, Hf, Sc) by the first principles. The calculated energy shows that Al_3_Zr has the lowest formation enthalpies and is the easiest to form.

However, few systematic investigations have been performed regarding the mechanical anisotropy of trialuminides Al_3_TM (TM, i.e., Sc, Ti, V, Y, Zr, Nb, La, Hf, Ta). Particularly, up to now, the first-principles study of work function of these trialuminides Al_3_TM phases is almost blank. Accordingly, it is interesting to research the differences and similarities in elastic properties when the Al element forms a bond with those transition elements. Therefore, further attempts in this respect still need to research the properties of D0_22_-type trialuminides.

Indeed, when the properties of compounds are to be explored and cannot be measured by experimental means, theoretical simulation is a very effective method to forecast the properties of materials. Faithfully, the first principles calculation method based on the density functional theory (DFT) is a very powerful way to accurately study the physical properties of compounds [26,27]. Consequently, the structure, mechanical anisotropy, electronic properties, and work function of trialuminides Al_3_TM (TM, i.e., Sc, Ti, V, Y, Zr, Nb, La, Hf, Ta) have been systematically explored by first-principles calculation method in the present work. Based on the simulation results, an experiment was designed to verify the simulation results. The research results can supply more theoretical and technological guidance on D0_22_-type trialuminides design.

## 2. Methods and Details

In order to more systematically study the internal mechanisms of D0_22_ structure Al_3_TM (TM = Sc, Ti, V, Y, Zr, Nb, La, Hf, Ta), the first-principle calculations based on density functional theory are implemented in the CASTEP (Cambridge Serial Total Energy Package) code [28]. The generalized gradient approximation (GGA) method is applied to the exchange correlation function, and three parameterization ways, Perdew, Burke, and Ernzerhof (PBE) are adopted [29,30]. The 3s^2^3p^1^, 3s^2^3d^1^4s^2^, 3s^2^3p^6^3d^2^4s^2^, 3s^2^3p^6^3d^3^4s^2^, 4d^1^5s^2^, 4s^2^4p^6^4d^2^5s^2^, 4s^2^4p^6^4d^4^5s^1^, 5s^2^5p^6^5d^1^6s^2^, 5d^2^6s^2^ and 5d^3^6s^2^ electrons are explicitly considered as valence for Al, Sc, Ti, V, Y, Zr, Nb, La, Hf and Ta, respectively. In the process of optimization, the total energy of the self-consistent convergence condition is less than 1.0 × 10^−5^ eV/atom. The maximum displacement is 0.001 Å and the maximum force acting on each atom is 0.03 eV/Å. The maximum stress deviation is 0.05 GPa, while the SCF convergence accuracy is set to 5.0 × 10^−7^ eV/atom. Using plane wave basis set, the cutoff energy is 500 eV, while the total energy of this work will eventually converge to less than 1 meV. Based on the characteristics of D0_22_ crystal type in this study, the k-point grid of energy integration in irreducible Brillouin region is generated by using the Monkhorst-Pack method. A 22 × 22 × 10 k-point mesh for the static calculation is used for this work.

Based on the simulation results, we designed an experiment to verify the simulation results. A high entropy alloy reinforced by D0_22_-type Al_3_Ta phase was designed. Several elements of Co, Cr, Fe, Ni and Ta powders with purity of 99.96% were used as designed materials (Co 21.6 ± 0.2, Cr 19.8 ± 0.3 Fe 21.5 ± 0.2 Al 35.5 ± 0.3 Ta 1.6 ± 0.2 with atomic percentage). These nearly pure powders were first ball-ground and then pressed into shape. Finally, it is sintered in a vacuum tube furnace. To ensure uniformity, the alloy is smelted five times and then soldered at 1200 °C. At last, the X-ray diffraction (XRD) was tested at room temperature and operated at 30 kW, 50 mA, with Cu Kα radiation. The instrument model was D/max-2550 X-ray diffractometer (Rigaku inc, Tokyo, Japan). The data of XRD were analyzed with JADE software (version 6.5, Jade, Christchurch, New Zealand). At the same time, the samples were characterized by scanning electron microscope (SEM SU6600, Hitachi High-Technology Corp., Tokyo, Japan) and transmission electron microscope (TEM JEOL 2100 F, JEOL Co., Ltd., Tokyo, Japan).

## 3. Results and Discussion

### 3.1. Structural Properties and Stability

**Figure 1:**
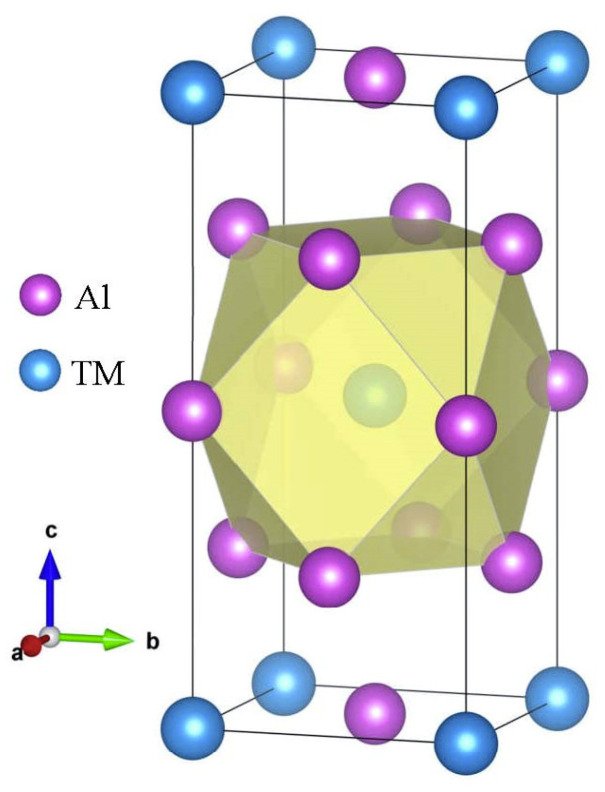
The crystal structure of D0_22_-type Al_3_TM compounds.

Firstly, the structural stability of these D0_22_-type compounds was investigated. The crystal structure of the binary compound D0_22_-Al_3_TM, where TM is Sc, Ti, V, Y, Zr, Nb, La, Hf, Ta, is displayed in Figure 1. The D0_22_-type compounds are tetragonal crystal with a space group of I4/mmm. Except for Al element, the composition of these compound elements is mainly composed of transition elements. Although these transition elements form the same crystal structure with aluminum, they have a greater impact on the properties of the compound. Being able to understand their internal mechanisms is very important for the design of alloys.

In order to determine the phase stability of Al_3_TM (TM = Sc, Ti, V, Y, Zr, Nb, La, Hf, Ta) intermetallics, the formation enthalpy and cohesive energy of these compounds are calculated by following equations [31,32]:

(1) $$∆H=\frac{1}{x+y}\;(E_{\mathrm{tot}}-{\mathrm{xE}}_{\mathrm{solid}}^{A}-{\mathrm{yE}}_{\mathrm{solid}}^{B}),$$

(2) $$E_{\mathrm{coh}}=\frac{1}{x+y}\;(E_{\mathrm{tot}}-{\mathrm{xE}}_{\mathrm{atom}}^{A}-{\mathrm{yE}}_{\mathrm{atom}}^{B}),$$

where ∆H is the formation enthalpy of Al_3_TM compound. $E_{\mathrm{tot}}$ is the total energy of Al_3_TM phase. $E_{\mathrm{solid}}^{A}$ and $E_{\mathrm{solid}}^{B}$ are the energy of Al and TM atom, respectively. In addition, $E_{\mathrm{coh}}$ is the cohesive energy of Al_3_TM compound. $E_{\mathrm{atom}}^{A}$ and $E_{\mathrm{atom}}^{B}$ is the energy of Al and TM free atom, respectively. The x and y are the number of Al and TM atom in the D0_22_-Al_3_TM crystal structure, respectively. The calculated results of this work are noted in Table 1 and compared with other previous values [13,21,32,33]. The order of the compounds is arranged according to the atomic number of the TM atom in the periodic table of the elements. After analysis, it is evidence that the obtained results are basically coincident with the other calculated values, which indicates the reliability and good self-consistency of the proposed method.

The first principle calculation is carried out at the ground state of 0 K and 0 Pa. The formation enthalpy of the compound is negative, indicating that the formation of the compound is an exothermic process. The more negative the formation enthalpy, the more stable the compound is. It can be seen from Table 1 that the formation enthalpy of these nine D0_22_-type compounds is less than zero, meaning that these compounds are thermodynamically stable in the ground state.

**Figure 2:**
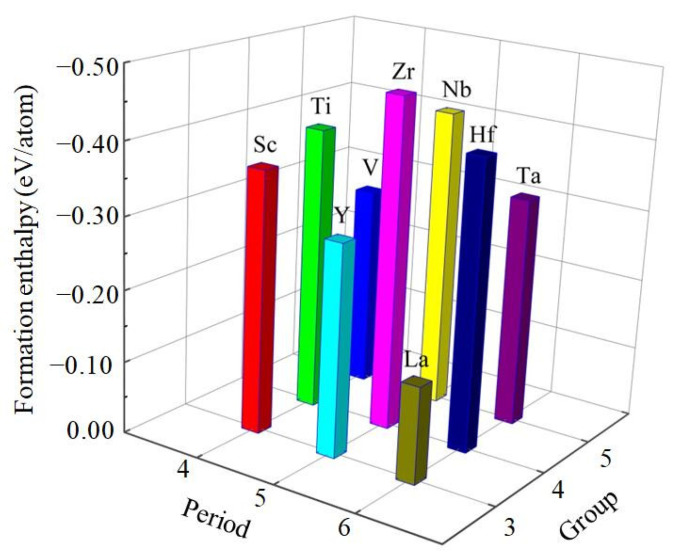
The calculated formation enthalpies of Al_3_TM compounds.

It is clear from Table 1 that the calculated value of cohesive energy of these compounds is −4.221, −4.793, −4.846, −4.178, −4.916, −5.473, −3.934, −4.906, −5.151 eV/atom for Al_3_Sc, Al_3_Ti, Al_3_V, Al_3_Y, Al_3_Zr, Al_3_Nb, Al_3_La, Al_3_Hf, Al_3_Ta, respectively. Generally speaking, the large cohesive energy of compounds can only show that the free atoms of the two elements release more energy when they bond. If the energy needed to break the combination of the two elements is also large, the compound is still relatively unstable. However, the thermodynamic stability of compounds is affected by their enthalpy of formation. To some extent, the lower the enthalpy of formation, the easier the compounds are to form and the higher the thermodynamic stability [35,36]. The results show that the value of formation enthalpy of these D0_22_-type compounds is −0.366, −0.396, −0.283, −0.295, −0.464, −0.419, −0.134, −0.406, −0.322 eV/atom for Al_3_Sc, Al_3_Ti, Al_3_V, Al_3_Y, Al_3_Zr, Al_3_Nb, Al_3_La, Al_3_Hf, Al_3_Ta, respectively. The formation enthalpy of Al_3_Zr is the lowest, which suggests that Al_3_Zr alloy has the strongest formation ability and the most stable thermodynamics, while Al_3_La is on the contrary. Therefore, the alloying ability of the nine D0_22_-type compounds from strong to weak can be arranged as Al_3_Zr > Al_3_Nb > Al_3_Hf > Al_3_Ti > Al_3_Sc > Al_3_Ta > Al_3_Y > Al_3_V > Al_3_La, as showed in Figure 2. Considering all these compounds together, it is found that the Al_3_Zr is the most thermodynamic stable compound.

### 3.2. Mechanical Stability, Elastic Properties and Moduli

As well known, elastic constant is a very significant index to characterize the properties of compounds. The elastic constant is the index of material elasticity, which is related to the stress-strain relationship in the anisotropic medium. To some extent, the elastic constant also indicates the influence of crystal dynamics on mechanical behavior. The stress-strain method is used to calculate the elastic constants in the present calculation process and the results can be listed in Table 2. Furthermore, for the D0_22_-type crystal, tetragonal phase (C_11_, C_33_, C_44_, C_66_, C_12_, and C_13_) [37], the elastic constants can be restricted by the following Formula (3):

C_11_ > 0, C_33_ > 0, C_44_ > 0, C_66_ > 0 (C_11_ − C_12_) > 0, (C_11_ + C_33_ − 2C_13_) > 0, [2(C_11_ + C_12_) + C_33_ + 4C_13_] > 0. (3)

**Figure 3:**
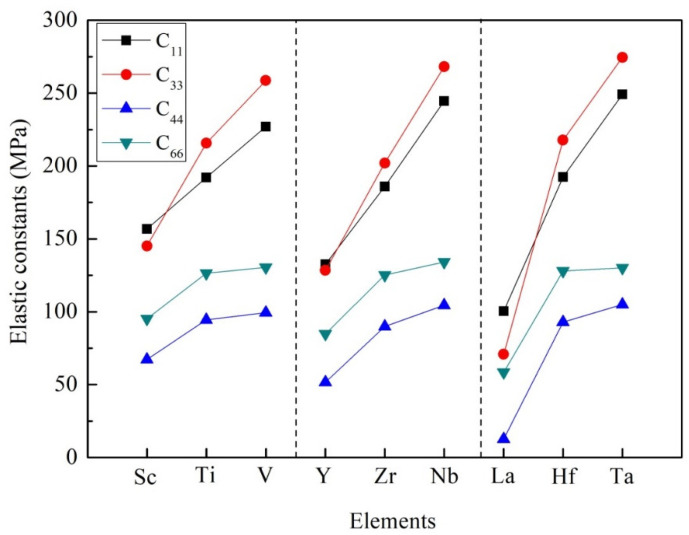
The trend of elastic constants of Al_3_TM compounds.

It is noticeable that the elastic constants calculated in Table 2 conform to the mechanical stability criterion. According to Equation (3), these D0_22_-type compounds are mechanically stable at 0 K. The elastic constants C_11_, C_22_, and C_33_ mean the compressibility of the crystal structure along the a-axis, b-axis, and c-axis, respectively. In the tetragonal system, C_11_ and C_22_ have the same value. Similarly, the value of C_44_ in the tetragonal system is the same as that of C_55_. The value of C_44_ indicates the ability to resist shear strain in (100) or (010) plane, while the value of C_66_ represents the ability to resist shear strain in (001) plane. In view of Al_3_Sc, Al_3_Y and Al_3_La compounds, the calculated value of C_33_ is less than that of C_11_, which proves that the a-axis has greater compression resistance than the c-axis. However, for other compounds, such as Al_3_Ti, Al_3_V, Al_3_Zr, Al_3_Nb, Al_3_Hf, Al_3_Ta, the elastic constants of C_33_ are higher than that of C_11_, suggesting that the c-axis has greater compression resistance than the a-axis, as illustrated in Figure 3. In particular, it was found that Al_3_Ta has the highest resistance along the a-axis, b-axis, and c-axis. Besides, the values of C_11_ and C_33_ are larger than that of C_44_ and C_66_. It means that these D0_22_-type phases are highly deformation resistant under uniaxial stress along the a- and c-axis.

On the other hand, the bulk modulus (B), shear modulus (G), Young’s modulus (E) and Poisson’s ratio (σ) of Al_3_TM crystal can be calculated by Viogt-Reuss-Hill (VRH) approximation method. Using the following formulas [38], the calculated values are shown in Table 3.

(4) $$B_{H}=\frac{1}{2}\left(B_{V}+B_{R}\right),$$

(5) $$G_{H}=\frac{1}{2}\left(G_{V}+G_{R}\right),$$

(6) $$E=\frac{9B_{H}G_{H}}{\left(3B_{H}+G_{H}\right)},$$

(7) $$\sigma =\frac{\left(3B_{H}-2G_{H}\right)}{\left[2\left(3B_{H}+G_{H}\right)\right]},$$

where the subscript symbols H denotes the modulus values obtained by the Hill approximation. The subscript V and R mean the modulus values obtained by the Voigt and Reuss approximation methods, respectively. Furthermore, the Voigt approximation limits the maximum of elastic modulus and the Reuss approximation is considered to be the minimum of elastic modulus. The Hill approximation uses the average value of Voigt and Reuss to express the elastic constants of materials.

**Figure 4:**
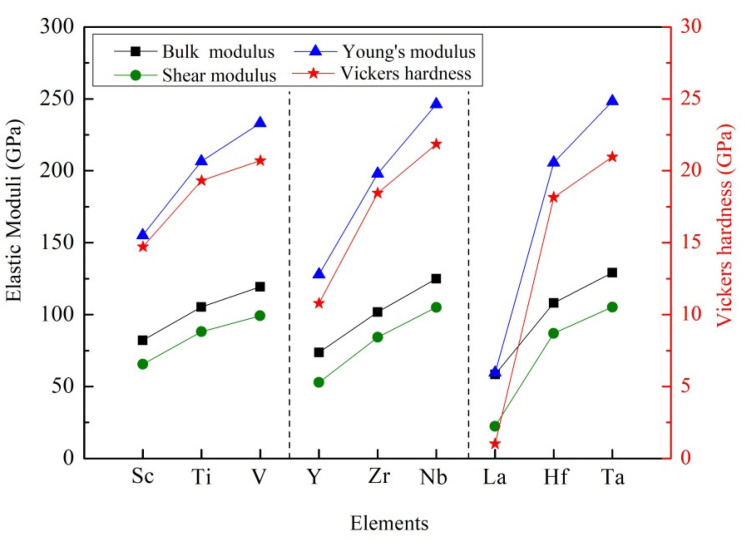
Comparison of moduli and Vickers hardness of different compounds.

It is noted that the calculated values of elastic moduli are summarized in the Table 3. Obviously, the bulk modulus of these D0_22_-type compounds decreased in order: Al_3_Ta > Al_3_Nb > Al_3_V > Al_3_Hf > Al_3_Ti > Al_3_Zr > Al_3_Sc > Al_3_Y > Al_3_La, as shown in Figure 4. The bulk modulus of crystal reflects the resistance of crystal under water pressure. At the microscopic level, the bulk modulus of the crystal is determined by the strength of the chemical bond. The larger the bulk modulus of the crystal, the stronger the chemical bond strength and the stronger the compression resistance. The calculated results show that Al_3_Ta has the strongest resistance of compression. Oppositely, Al_3_La has the weakest resistance of compression.

Generally, shear modulus refers to the ability of a material to resist shear strain. The higher the shear modulus, the stronger the rigidity of the material. As listed in Table 3, Al_3_Ta and Al_3_La have the largest shear modulus (108.19 GPa) and smallest one (22.41 GPa), respectively. At the same time, Young’s modulus is a term of material mechanics, which is used to express the deformation resistance of solid materials. The rigidity of the material can be reflected by the value of Young’s modulus. That is to say, the greater the Young’s modulus is, the greater the rigidity of the material is, and the harder it is to deform. It can be seen that Al_3_Ta and Al_3_La have the largest Young’s modulus (254.17 GPa) and smallest one (59.62 GPa). It is evident that Al_3_Ta has the greater rigidity and is difficult to deform.

Furthermore, the brittleness and ductility of the materials can be judged by Poisson’s ratio. This is due to Poisson’s ratio being the ratio of transverse strain to longitudinal strain when the material is deformed under tension or compression. A value of 0.26 is taken as the critical value of brittle and plastic separation. When the value of Poisson’s ratio is greater than 0.26, it can be considered as ductile material. On the contrary, it can be determined as brittle material.

When the value of Poisson’s ratio is less than 0.26, it can be judged as brittle material. Otherwise, it can be judged as ductile material [39,40]. As for these D0_22_-type compounds, the Poisson’s ratios are 0.185, 0.173, 0.175, 0.211, 0.176, 0.172, 0.183, and 0.175 for Al_3_Sc, Al_3_Ti, Al_3_V, Al_3_Y, Al_3_Zr, Al_3_Nb, Al_3_Hf, and Al_3_Ta respectively, while the Poisson’s ratio of Al_3_La is 0.330. Therefore, it is noted that Al_3_La shows toughness and the other eight compounds exhibit brittle property. Similarly, brittleness and ductility of compounds can be predicted by the ratio of bulk modulus to shear modulus [41,42]. Based on the Pugh standard, the material presents brittleness when the value of B/G is less than 1.75, otherwise the material exhibit toughness. As can be shown in Table 3 that the B/G of Al_3_Sc, Al_3_Ti, Al_3_V, Al_3_Y, Al_3_Zr, Al_3_Nb, Al_3_Hf, and Al_3_Ta is less than 1.75, indicating that these eight D0_22_-type compounds are brittle. On the contrary, the B/G value of Al_3_La is greater than 1.75, which shows toughness.

Ultimately, it is reported that the hardness of a compound is directly related to its shear modulus and Young’s modulus. At present, although the accurate relationship between hardness and elastic modulus has not been determined, the larger elastic modulus can represent the higher hardness. For the nine compounds studied, their Vickers hardness (H_V_) of Al_3_TM-type compounds can be forecasted by the following empirical formula [43]:

(8) $$\mathrm{Hv}=2{\left(G^{3}/B^{2}\right)}^{0.585}-3,$$

As presented in Table 3, the maximum Vickers hardness of Al_3_TM is Al_3_Ta, which is 21.94 GPa. The minimum Vickers hardness is Al_3_La and its value is 1.02 GPa. Similar trends indicate that Al_3_Ta and Al_3_La have the largest Young’s modulus and smallest one, as indicated in Figure 4. It is also confirmed that Young’s modulus has a decisive effect on the hardness of the compound.

### 3.3. Mechanical Anisotropy

It is very important to study the anisotropy of D0_22_-type compounds since this index has influence on the macroscopic mechanical properties of the alloy. In the present calculation, the universal anisotropic index (A^U^), the percent anisotropy index (A_B_ and A_G_) and the shear anisotropic factors (A_1_, A_2_ and A_3_) are estimated by the following expressions [44]:

(9) $$A^{U}=5\frac{G_{V}}{G_{R}}+\frac{B_{V}}{B_{R}}-6\geq 0,$$

(10) $$A_{B}=\frac{B_{V}-B_{R}}{B_{V}+B_{R}}\times 100\%,$$

(11) $$A_{G}=\frac{G_{V}-G_{R}}{G_{V}+G_{R}}\times 100\%,$$

(12) $$A_{1}=\frac{4C_{44}}{C_{11}+C_{33}-2C_{13}}\;\mathrm{for}\;(100)\;\mathrm{plane},$$

(13) $$A_{2}=\frac{4C_{55}}{C_{22}+C_{33}-2C_{23}}\;\mathrm{for}\;(010)\;\mathrm{plane},$$

(14) $$A_{3}=\frac{4C_{66}}{C_{11}+C_{22}-2C_{12}}\;\mathrm{for}\;(001)\;\mathrm{plane}.$$

The generated universal anisotropic index (A^U^), percent anisotropy (A_B_ and A_G_) and shear anisotropic factors (A_1_, A_2_ and A_3_) of these D0_22_-type compounds are exhibited in Table 4. The anisotropy of D0_22_-type compounds can be directly reflected by the value of A^U^. In case of A^U^ is equal to zero, the crystal is isotropic. The larger the value of A^U^, the stronger the anisotropy, and vice versa. It is noted that the values of A^U^ of Al_3_Sc, Al_3_Ti, Al_3_V, Al_3_Y, Al_3_Zr, Al_3_Nb, Al_3_La, Al_3_Hf, Al_3_Ta are 0.257, 0.400, 0.220, 0.385, 0.455, 0.177, 1.927, 0.440, and 0.126, respectively. In this case, the anisotropic properties of these D0_22_-type compounds can be listed as Al_3_La > Al_3_Zr > Al_3_Hf > Al_3_Ti > Al_3_Y > Al_3_Sc > Al_3_V > Al_3_Nb > Al_3_Ta.

When the values of A_B_ and A_G_ are not equal to zero, it shows that the bulk modulus and shear modulus of the crystal are anisotropic. The values of A_B_ and A_G_ correspond to the anisotropy. It reveals that Al_3_La has the largest A_B_ value 2.839, indicating that the bulk modulus anisotropy of Al_3_La is the strongest. Then, the bulk modulus anisotropy of Al_3_Y, Al_3_Sc, Al_3_Zr, Al_3_Nb, Al_3_Ti, Al_3_Hf, Al_3_V, and Al_3_Ta decrease gradually. Evidently, The A_G_ value of Al_3_La is the highest and that of Al_3_Ta is the lowest, indicating that Al_3_La and Al_3_Ta have the strongest and weakest shear modulus anisotropy, respectively.

Furthermore, the shear factors A_1_, A_2_, and A_3_ can be used to represent anisotropy on (100), (010), and (001) planes. When the values of A_1_, A_2_, and A_3_ are 1, the crystal is isotropic. According to the calculated results, all D0_22_-type compounds have different degrees of elastic anisotropy, as noted in the Table 4. Among these D0_22_-type phases, the A_1_ (A_2_) values of Al_3_La deviate most severely from 1, which imply that Al_3_La exhibits the strongest shear anisotropy in (100) and (010) planes. Conversely, the values of A_1_ (A_2_) of Al_3_Ta have the least deviation among these D0_22_-type compounds, which mean that the Al_3_Ta exists the lowest shear anisotropy in the (100) plane and (010) plane. The A_3_ value of Al_3_La has the most deviation from 1, hinting that the Al_3_La shows the highest shear anisotropy in the (001) plane. These D0_22_-type phases all have a wide deviation from 1 in the (001) plane.

On the other hand, the three-dimensional (3D) image has the characteristics of clear hierarchy and visual intuition, which can show the anisotropic characteristics of these D0_22_-type compounds more clearly and vividly. In this study, bulk modulus, shear modulus, and Young’s modulus of different D0_22_-type compounds are drawn with spherical coordinates. The relationship between bulk modulus, shear modulus, Young’s modulus, and different directions can be realized by the following formulas [45,46]:

(15) $$\frac{1}{B}=\left(S_{11}+S_{12}+S_{13}\right)\left(l_{1}^{2}+l_{2}^{2}\right)+\left(2S_{13}+S_{33}\right)l_{3}^{2},$$

(16) $$\frac{1}{G}=\frac{1}{2}\left(S_{66}+S_{44}\right)+\left(4S_{11}-4S_{12}-2S_{66}\right)l_{1}^{2}l_{2}^{2}+\left(2S_{11}+2S_{33}-4S_{13}-2S_{44}\right)\left(l_{1}^{2}l_{3}^{2}+l_{2}^{2}l_{3}^{2}\right)+\frac{1}{2}\left(S_{44}-S_{66}\right)l_{3}^{2},$$

(17) $$\frac{1}{E}=S_{11}\left(l_{1}^{4}+l_{2}^{4}\right)+\left(2S_{13}+S_{44}\right)\left(l_{1}^{2}l_{3}^{2}+l_{2}^{2}l_{3}^{2}\right)+S_{33}l_{3}^{4}+\left(2S_{12}+S_{66}\right)l_{1}^{2}l_{2}^{2},$$

where, $l_{1}=sin\theta cos\phi$, $l_{2}=sin\theta sin\phi$, $l_{3}=cos\theta$, *l*_1_, *l*_2_ and *l*_3_ the directional cosines, *S_ij_* the elastic compliance constants. If the system is isotropic, the three-dimensional directional correlation is spherical. The deviation of spherical shape suggests the degree of anisotropy.

**Figure 5:**
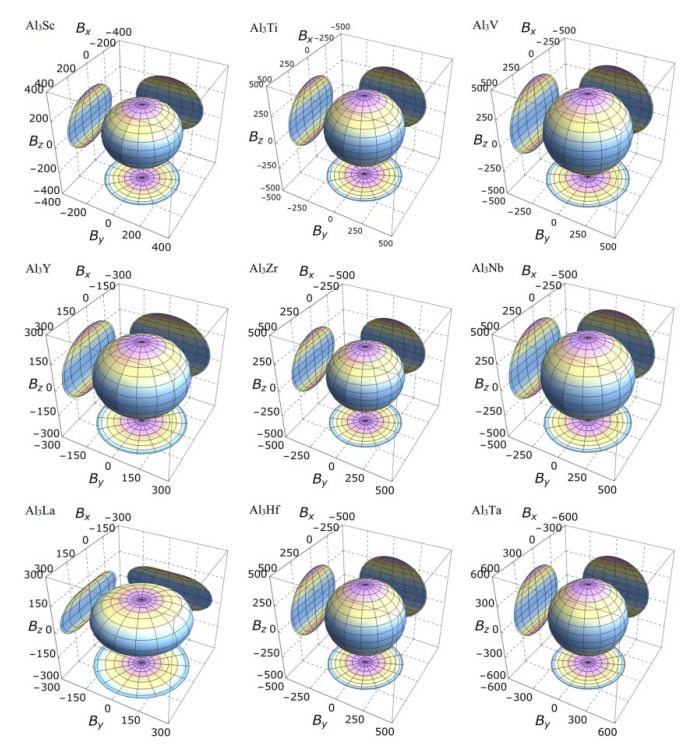
The anisotropic characteristics of bulk modulus of the Al_3_TM compounds.

**Figure 6:**
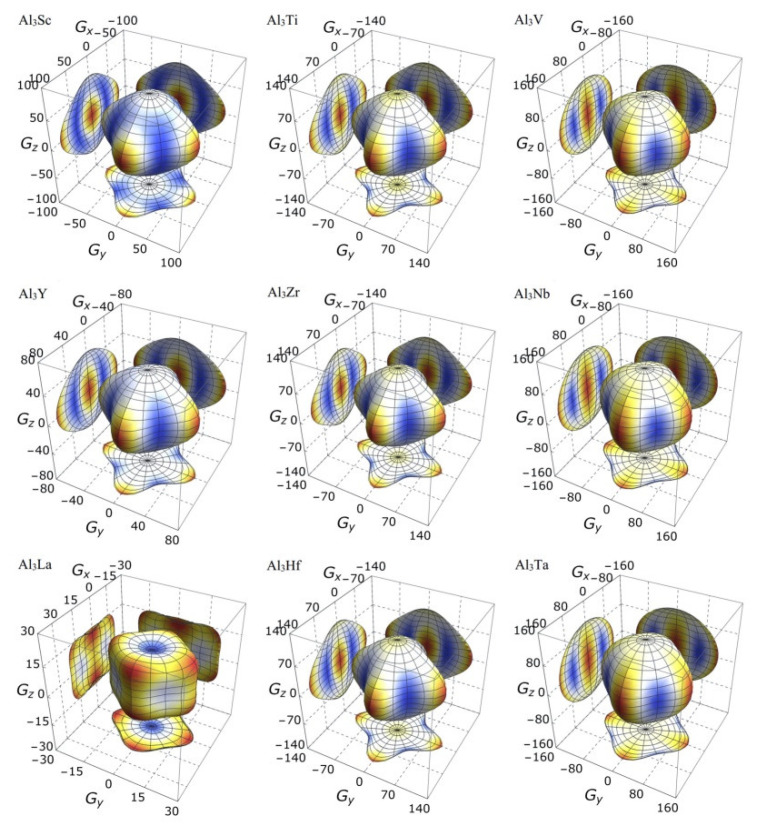
The anisotropic characteristics of shear modulus of the Al_3_TM compounds.

**Figure 7:**
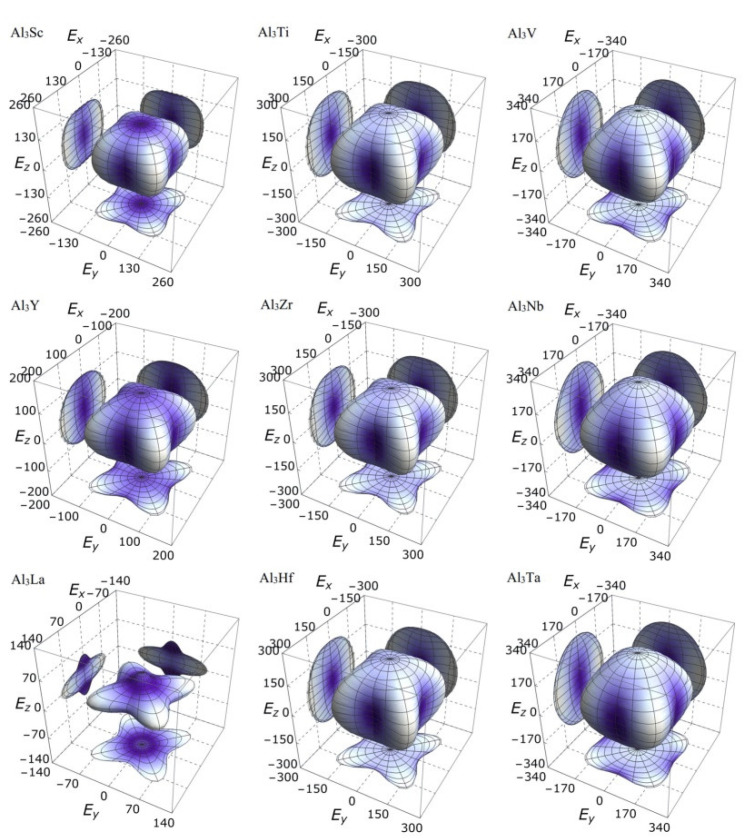
The anisotropic characteristics of Young’s modulus of the Al_3_TM compounds.

It is clear from the 3D stereoscopic pictures of bulk modulus, shear modulus, and Young’s modulus that D0_22_-type compounds with the same crystal structures and different compositions reveal different degree anisotropies. It can also be seen from Figure 5 that the bulk modulus of Al_3_La shows the strongest anisotropy. This result is consistent with the maximum anisotropy index A_B_ of Al_3_La calculated in Table 4. As proved in Figure 6, the shear modulus anisotropy of Al_3_La is the strongest, while that of Al_3_Ta is the weakest. This phenomenon is consistent with the results obtained by the anisotropy index A_G_ in Table 4. Finally, the anisotropic properties of Young’s modulus of these D0_22_-type compounds can be arranged as Al_3_La > Al_3_Zr > Al_3_Hf > Al_3_Ti > Al_3_Y > Al_3_Sc > Al_3_V > Al_3_Nb > Al_3_Ta, as noted in Figure 7.

**Figure 8:**
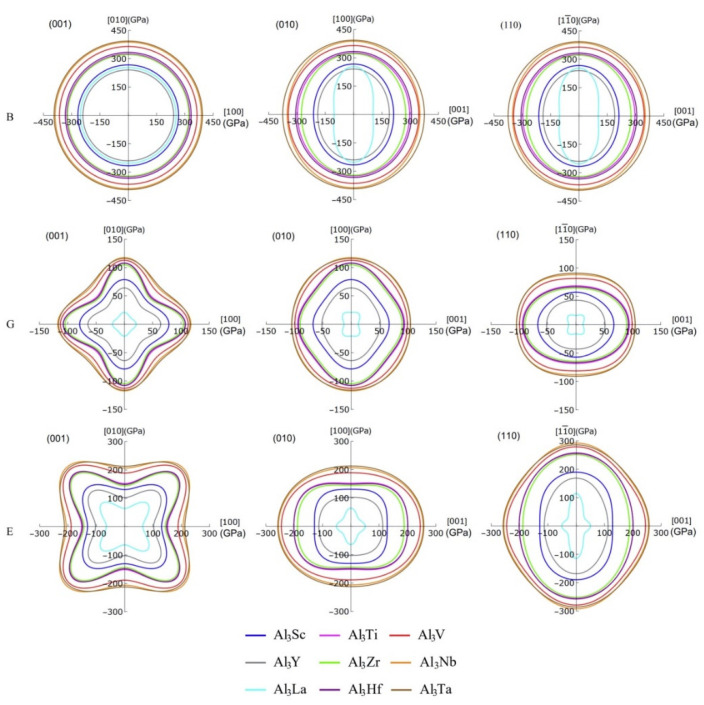
The projections of bulk modulus, shear modulus and Young’s modulus on the (001), (010), and (110) crystal planes of Al_3_TM compounds.

In particular, the projecting images of bulk modulus, shear modulus, and Young’s modulus can display the anisotropy details of the compounds more clearly, which are given in Figure 8. It should be noted that the projection features of (100) and (010) crystal planes are the same, so only the projections of (001), (010), and (110) plane are listed here. Obviously, the bulk modulus of these compounds shows greater anisotropic on the (010) and (100) planes, but it exhibits isotropy on the (001) plane. The degree of anisotropy of the same compound on (010) and (100) is the same, suggesting that the order of anisotropy is Al_3_La > Al_3_Y > Al_3_Sc > Al_3_Zr > Al_3_Nb > Al_3_Ti > Al_3_Hf > Al_3_V > Al_3_Ta. Additionally, the shear moduli of these D0_22_-type compounds are anisotropic on all three planes. Especially, the 3D diagram morphology of Al_3_La shear modulus anisotropy is different from that of the other eight compounds. These eight compounds have the similar anisotropy on the (001) crystal plane, while the anisotropy degree of Al_3_Ta displays the lowest. Furthermore, among these eight compounds, the anisotropy of Al_3_Ta in three crystal faces (001), (010), and (100) is smaller. Similarly, the Young’s moduli of the nine compounds are anisotropic in three planes. The degree of anisotropy of Al_3_Ti, Al_3_V, Al_3_Y, Al_3_Zr, Al_3_Nb, Al_3_Hf, and Al_3_Ta is smaller on (100) and (010) crystal planes, while that of Al_3_Sc and Al_3_La is larger on (100) and (010) crystal planes. However, it is interesting to note that the anisotropy of Young’s modulus is in the opposite direction to that of the shear modulus. Obviously, the Young’s modulus of Al_3_Ta is less anisotropic in all three planes, while the anisotropy of Al_3_La is greater.

Strangely, from Figure 5, Figure 6, Figure 7 and Figure 8, the anisotropic appearances of Al_3_La in bulk modulus, shear modulus, and Young’s modulus is quite different from those of the other eight compounds. As discussed earlier, Al_3_La has higher enthalpy of formation, lower modulus values, unique toughness, and smallest Vickers hardness. Thus, it is speculated that D0_22_-type Al_3_La may not exist.

Corresponding to Figure 8, the Table 5 summarizes more specific values of bulk modulus, shear modulus, and Young’s modulus in four directions. Due to the structural properties of D0_22_-type compounds, the bulk modulus, shear modulus, and Young’s modulus are equal in the directions of [100] and [010]. Likewise, the bulk modulus of these compounds in the $\left[1\bar{1}0\right]$ direction is equal to those in the [100] and [010] directions. However, the shear modulus and Young’s modulus of these compounds in the $\left[1\bar{1}0\right]$ direction are not equal to those in the [100] and [010] directions. The maximum and minimum bulk modulus of Al_3_Ta and Al_3_La in [001] direction is 375.16 and 103.00 GPa, respectively. This means that Al_3_Ta has the strongest resistance of compression in [001] direction. In addition, the shear modulus in the directions of [001] and $\left[1\bar{1}0\right]$ are smaller than those in the directions of [100] and [010], which indicates stronger shear strain capacity. The maximum shear modulus of Al_3_Nb in [100] and [010] direction is 117.56 GPa. Furthermore, the Young’s modulus of other compounds except Al_3_Sc and Al_3_La in the [001] direction is greater than that in the [100] and [010] directions. The reason is that the C_33_ value of Al_3_Sc and Al_3_La is smaller than C_11_ and C_22_, which leads to greater compressibility in c-axis direction.

### 3.4. Electronic Structures

**Figure 9:**
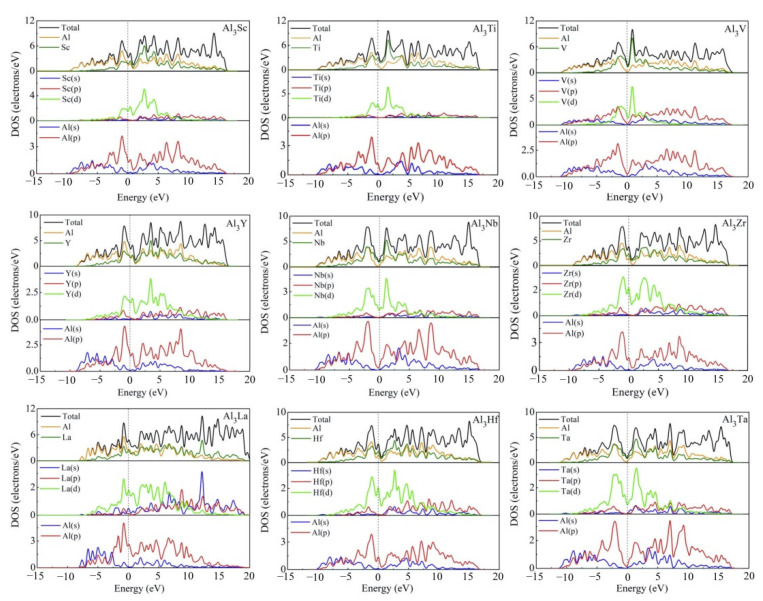
Total and partial electronic densities of states near Fermi level of Al_3_TM compounds.

The compounds in present work are all D0_22_-type compounds that are bound with transition metals. Because of their orientation-related bonding properties, it is of great significance to analyze the chemical bonding properties and electronic structures of these D0_22_-type compounds. In Figure 9, we calculated the total density of states (TDOS) and partial density of states (PDOS) of D0_22_-Al_3_TM compounds. The vertical dashed line at zero energy shows the Fermi level (E_F_). Obviously, the density of states at Fermi level is not zero, suggesting that these phases have metal properties and electrical conductivity. For Al_3_Sc, Al_3_Ti, Al_3_V, Al_3_Y, Al_3_Zr, Al_3_Nb, Al_3_La, Al_3_Hf, and Al_3_Ta phases, there is a peak near the Fermi level of the TDOS curves, implying their good conductivity.

It is obvious that the TDOS at the Fermi level for Al_3_Sc, Al_3_Ti, Al_3_V, Al_3_Y, Al_3_Zr, Al_3_Nb, Al_3_La, Al_3_Hf and Al_3_Ta are 3.43, 4.25, 1.99, 3.59, 3.85, 1.30, 4.65, 3.71, and 1.08 electrons/eV, respectively. The TDOS curve in Figure 9 also shows the pseudogap near E_F_, which is due to the electron transfer to the low energy region. The lower the position of E_F_ in the gap, the more stable the structure of intermetallics is. Thus, Al_3_Ta can be considered as the most stable compound. When the pseudogap is larger, it means stronger bond strength and higher deformation resistance. Compared with Al_3_V, Al_3_Nb, and Al_3_Ta in Figure 9, it can be noted that the pseudogap of Al_3_Ta has the widest gap, meaning that the covalent bond strength of Al_3_Ta is stronger than that of Al_3_V and Al_3_Nb. This explains why Al_3_Ta has the highest hardness. In view of nine compounds, the shapes of TDOS for Al_3_Sc, Al_3_Ti, Al_3_Y, Al_3_Zr, Al_3_La, and Al_3_Hf are similar, which demonstrate that their chemical bonds are similar. The TDOS curves of Al_3_V, Al_3_Nb, and Al_3_Ta are alike, which is to say that the V-shaped tip intersects the dotted line of Fermi level.

The PDOS curves clearly show that the contribution of Al-s on the surface of Fermi level is small, which is mainly contributed by the Al-p state. The Fermi level of TDOS is mainly formed by the strong hybridization of Al-p and TM (TM = Sc, Ti, V, Y, Zr, Nb, La, Hf, Ta)-d states. Obviously, the hybridization of Al-s and TM (TM = Sc, Ti, V, Y, Zr, Nb, La, Hf, Ta)-s states are less helpful for the Fermi level of TDOS. The obtained quantity of bonding electrons per atom between −13 eV and Fermi level is 3.005, 3.243, 3.493, 3.003, 3.242, 3.499, 2.997, 3.247, and 3.501 for Al_3_Sc, Al_3_Ti, Al_3_Y, Al_3_V, Al_3_Zr, Al_3_Nb, Al_3_La, Al_3_Hf and Al_3_Ta, respectively.

**Figure 10:**
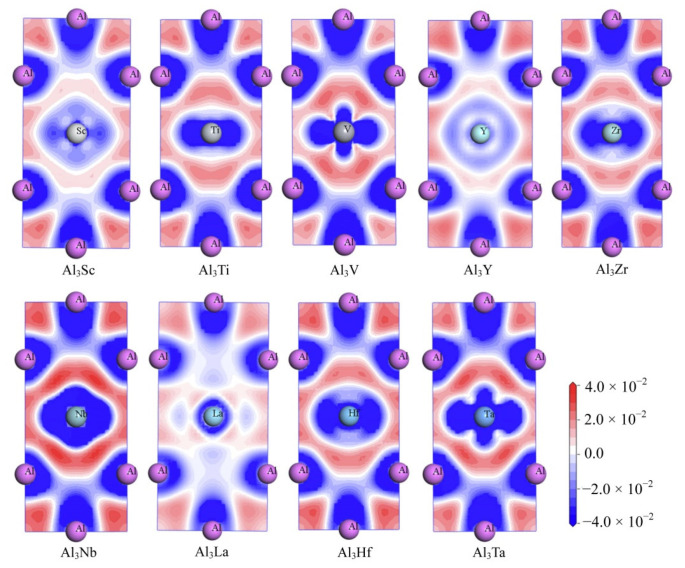
The electron density difference of Al_3_TM compounds.

Meanwhile, in order to explore the chemical bonds and charge transfer in all D0_22_-type compounds, the charge density difference on the (010) basal plane of these compounds were considered. In Figure 10, the distribution state of charge density difference of Al_3_Sc, Al_3_Ti, Al_3_V, Al_3_Y, Al_3_Zr, Al_3_Nb, Al_3_La, Al_3_Hf, and Al_3_Ta can be seen clearly. The values of charge density difference map are plotted from −0.04 to 0.04 e/Å^3^. Besides, the red and blue mean separately the aggregation and reduction of electrons.

As can be seen from the Figure 10, there are electron deletions around both Al and TM atoms (blue), and electron aggregation between Al and TM atoms (red). This suggests that the electrons are aggregated into a covalent Al-TM bond between the atoms Al and TM. Similarly, there is a large amount of charge accumulation between Al and Al atoms, indicating that the Al-Al bond in the compound has covalent bond characteristics. Furthermore, a larger degree of localization of electrons reflects a stronger bond. Consequently, the electron concentration between the Al and TM (V, Nb and Ta) atoms in Al_3_V, Al_3_Nb and Al_3_Ta compounds is higher, which leads to the conclusion that Al-TM (V, Nb and Ta) bonds are stronger. On the contrary, it can be suggested that the bonds of Al-La and La-La are the weakest, which is because the less the localization degree of electrons, the weaker the bonds are.

### 3.5. Work Function

**Figure 11:**
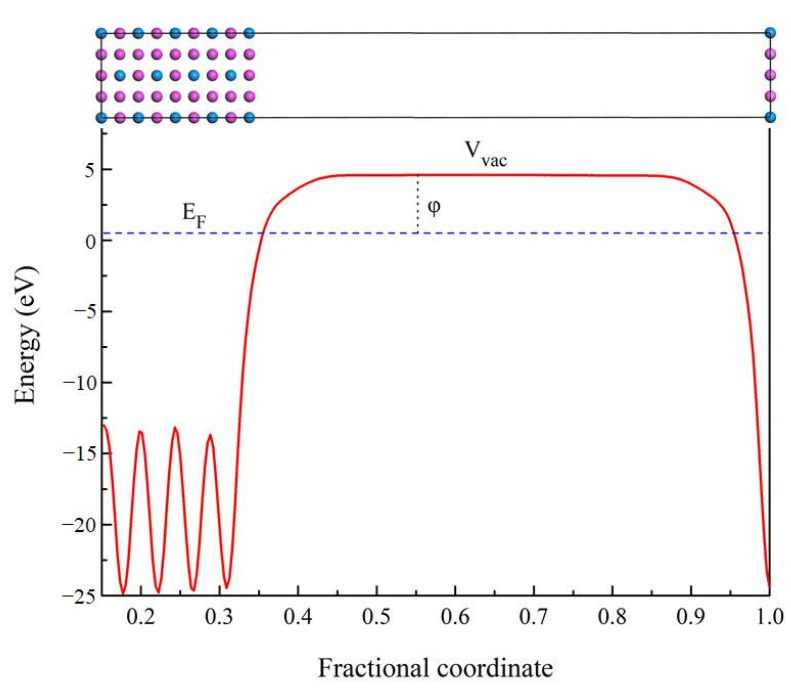
The schematic diagram of the electronic movement related to work function.

In order to further understand the properties of D0_22_-type compounds in this study, some surface work functions were calculated. Theoretically, the work function is the energy barrier used to move electrons from the surface of solid compounds to the free space, as noted by the following expression [47,48]:

(18) $$\varphi =V_{\mathrm{vac}}-E_{F},$$

where φ is work function, and symbol V_vac_ means the electrostatic potential of the vacuum region near the surface. E_F_ corresponds to the Fermi energy of the slab. The schematic diagram of the electronic movement is presented in Figure 11. When a compound has a higher electron work function, it takes more energy to change its electron state. Therefore, there are greater obstacles to improving the state or properties of compounds such as mechanical and elastic properties. The higher electron work function of the compound indicates that it has stronger atomic bond [49,50]. In present work, the work function value of Al_3_Sc, Al_3_Ti, Al_3_V, Al_3_Y, Al_3_Zr, Al_3_Nb, Al_3_La, Al_3_Hf, and Al_3_Ta is 3.838, 4.305, 4.206, 3.754, 3.953, 4.100, 3.291, 4.079, and 4.078 eV on (100) plane, respectively. It is found that the value of Al_3_La is the smallest. Therefore, the Young’s modulus and Vickers hardness of Al_3_La are the lowest, which is consistent with the previous calculated results.

### 3.6. Experimental Design

**Figure 12:**
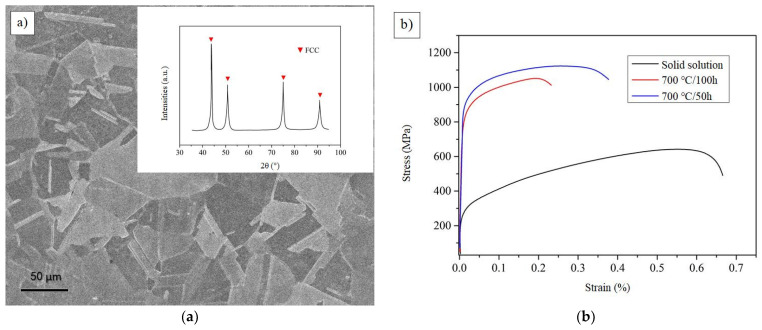
The SEM, XRD, and stress-strain curves of CoCrFeAlTa_0.16_. (**a**) The SEM and XRD images at 50 h annealing state; (**b**) The stress-strain curves of different heat treatment states.

Figure 12a shows the SEM and XRD images of representative CoCrFeAlTa0.16 HEA at 50 h annealing state. It is obvious that the alloy exhibits the crystal structure of FCC. The grain distribution in the image is clearly angular. The typical stress-strain curves of the solid solution and different heat treatment states can be seen in Figure 12b. It can be observed that the strength and toughness of the sample is better when it is treated at 700 °C/50 h. However, the strength and toughness of the samples were reduced when they were treated for 700 °C/100 h. It is possible that the grain size will be further enlarged as the solution time increases. In fact, the specimen annealed for 50 h will have D0_22_-type Al_3_Ta phase, resulting in its tensile strength up to 1120 MPa and elongation up to 26%. This result fully reflects the strengthening effect of Al_3_Ta phase.

**Figure 13:**
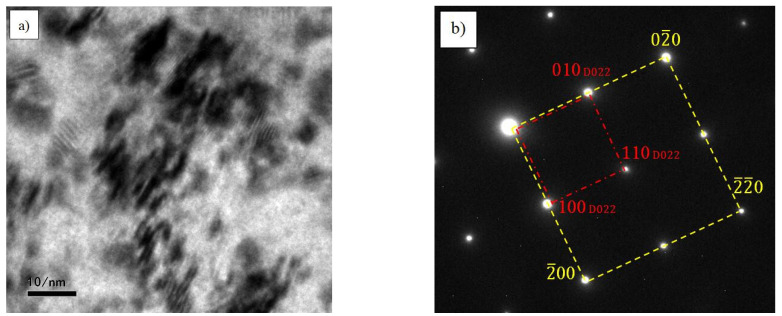

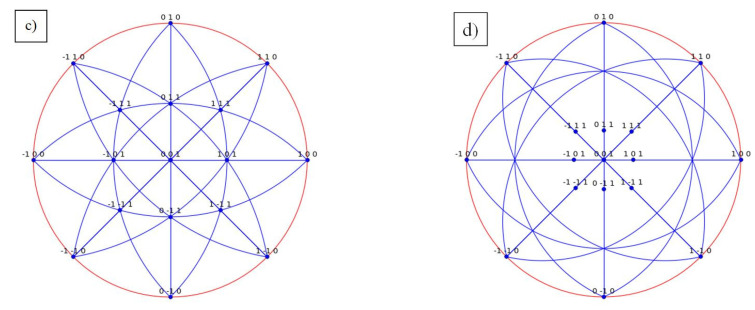
The TEM structure of D0_22_-Al_3_Ta phase. (**a**) the morphology of D0_22_-Al_3_Ta phase; (**b**) the diffraction pattern of D0_22_-Al_3_Ta phase; (**c**) the stereographic projection of the matrix; (**d**) the stereographic projection of D0_22_-Al_3_Ta phase.

In order to more clearly characterize the microstructure of D0_22_, Figure 13 exhibits the TEM structure of Al_3_Ta phase. According to the image, the Al_3_Ta phase is needle like and has a certain orientation. It presents a crystallographic relationship with the matrix, [001]_D022_//[001]_Matrix_. The diffraction pattern in Figure 13b clearly illustrates this relationship. It can see that plane $(\bar{2}\bar{2}$0) of the matrix corresponds to plane (110) of D0_22_-type Al_3_Ta phase. Moreover, stereographic projection of the orientation relationship denotes the crystal plane correspondence between the two phases more clearly, as shown in Figure 13c,d. This orientation relationship further indicates that the formation of Al_3_Ta contributes to the improvement of its strength and toughness. Therefore, the reliability of the simulation is further confirmed by the experiments, which has important reference values for the design and application of this material.

## 4. Conclusions

Overall, first principles calculations have been performed on elastic anisotropy, electronic structures, and work function of D0_22_-type Al_3_TM (TM = Sc, Ti, V, Y, Zr, Nb, La, Hf, Ta), including Al_3_Sc, Al_3_Ti, Al_3_V, Al_3_Y, Al_3_Zr, Al_3_Nb, Al_3_La, Al_3_Hf, and Al_3_Ta, respectively. The obtained results agree with the existing theoretical values.

It is noted that the thermodynamic properties of the nine D0_22_-type compounds are stable. The alloying ability of the nine D0_22_-type compounds from strong to weak can be arranged as Al_3_Zr > Al_3_Nb > Al_3_Hf > Al_3_Ti > Al_3_Sc > Al_3_Ta > Al_3_Y > Al_3_V > Al_3_La. The order of the bulk modulus of these D0_22_-type compounds is Al_3_Ta > Al_3_Nb > Al_3_V > Al_3_Hf > Al_3_Ti > Al_3_Zr > Al_3_Sc > Al_3_Y > Al_3_La. Specifically, Al_3_Ta and Al_3_La have the largest shear modulus (108.19 GPa) and smallest one (22.41 GPa) separately. It can be found that Al_3_Ta and Al_3_La have the largest Young’s modulus (254.17 GPa) and smallest one (59.62 GPa). It is evident that Al_3_Ta has the higher hardness and is not easy to deform.

Furthermore, the universal anisotropy of these D0_22_-type compounds can be listed as Al_3_La > Al_3_Zr > Al_3_Hf > Al_3_Ti > Al_3_Y > Al_3_Sc > Al_3_V > Al_3_Nb > Al_3_Ta. The Al_3_La and Al_3_Ta have the highest and lowest A_G_ values, demonstrating that the shear modulus anisotropy of Al_3_La is the strongest, while that of Al_3_Ta is the weakest. The A_3_ value of Al_3_La has the most deviation from 1, indicating that the Al_3_La shows the highest shear anisotropy in the (001) plane. Except for Al_3_Sc and Al_3_La, the Young’s modulus of other compounds in [001] direction is greater than that in [100] and [010] directions. From the standpoint of anisotropy, it is speculated that D0_22_-type Al_3_La may not exist.

It can be suggested that the density of states at Fermi level is not zero, suggesting that these phases have metal properties and electrical conductivity. Obviously, the pseudogap of Al_3_Ta has the widest width, meaning that the covalent bond strength of Al_3_Ta is stronger than that of Al_3_V and Al_3_Nb. The electron concentration between the Al and TM (V, Nb and Ta) atoms in Al_3_V, Al_3_Nb and Al_3_Ta compounds is higher, which shows that Al-TM (V, Nb and Ta) bonds are stronger. Ultimately, the work function of Al_3_La is the smallest on (100), demonstrating that the Young’s modulus and Vickers hardness of Al_3_La are the lowest.

Based on the calculated results, a kind of D0_22_ reinforced alloy was designed, which proves that this phase has an excellent strengthening effect. The matrix is strengthened by D0_22_-type Al_3_Ta phase, resulting in its tensile strength up to 1120 MPa and elongation up to 26%. The crystallographic relationship between D0_22_-type Al_3_Ta phase and matrix is [001]_D022_//[001]_Matrix_. The experimental results of this work further verify the reliability of the calculated results, which has important reference values for the design and application of the materials.

## Figures and Tables

**Table 1:** The calculated lattice constant (a, c in Å), density (*ρ*, g/cm^3^), volume (Å^3^), ∆Hr (eV/atom) and Ecoh (eV/atom) of the Al_3_TM compounds.

Species	Atomic Number	a	c	ρ	V	∆H	∆E
Al_3_Sc	21	4.024	8.840	2.920	143.173	−0.366	−4.221
		4.021 [13]	8.822 [13]				
Al_3_Ti	22	3.850	8.630	3.346	127.892	−0.396	−4.793
		3.851 [13]	8.576 [13]				
Al_3_V	23	3.773	8.324	3.697	118.484	−0.283	−4.846
		3.766 [13]	8.312 [13]				
		3.773 [21]	8.325 [21]		118.510 [21]	−0.277 [21]	
Al_3_Y	39	4.194	9.260	3.464	162.863	−0.295	−4.178
Al_3_Zr	40	3.963	9.027	4.034	141.757	−0.464	−4.916
Al_3_Nb	41	3.854	8.641	4.497	128.382	−0.419	−5.473
		3.855 [21]	8.645 [21]		128.470 [21]	−0.409 [21]	
Al_3_La	57	4.454	9.240	3.984	183.279	−0.134	−3.934
Al_3_Hf	72	3.946	8.918	6.206	138.842	−0.406	−4.906
		3.946 [33]	8.924 [33]		138.900 [33]	−0.404 [33]	−5.040 [33]
Al_3_Ta	73	3.862	8.591	6.789	128.121	−0.322	−5.151
		3.857 [34]	8.598 [34]			−0.318 [34]	

**Table 2:** The calculated elastic constants Cij (GPa) of Al_3_TM compounds.

Species	C_11_	C_12_	C_13_	C_33_	C_44_	C_66_
Al_3_Sc	156.86	56.88	42.17	145.14	67.20	95.17
	158.00 [13]	60.00 [13]	42.00 [13]	158.00 [13]	63.00 [13]	93.00 [13]
Al_3_Ti	192.06	84.53	44.60	215.66	94.46	126.42
	192.00 [13]	84.00 [13]	49.00 [13]	216.00 [13]	94.00 [13]	122.00 [13]
Al_3_V	227.01	89.03	45.95	258.69	99.37	130.55
	233.00 [13]	77.00 [13]	47.00 [13]	258.00 [13]	104.00 [13]	129.00 [13]
	220.87 [21]	92.69 [21]	45.26 [21]	256.95 [21]	98.57 [21]	130.25 [21]
Al_3_Y	132.51	58.63	38.77	128.45	51.60	84.84
Al_3_Zr	185.96	85.34	43.13	202.08	90.00	125.22
Al_3_Nb	244.54	92.28	45.94	268.13	104.57	134.22
	242.57 [21]	92.85 [21]	45.78 [21]	266.84 [21]	102.41 [21]	134.03 [21]
Al_3_La	100.51	55.51	39.49	70.85	12.67	58.57
Al_3_Hf	192.46	88.32	48.29	217.76	92.91	127.96
	193.60 [33]	87.10 [33]	47.40 [33]	217.80 [33]	92.40 [33]	123.30 [33]
Al_3_Ta	249.05	88.99	52.89	274.55	104.96	130.05

**Table 3:** The calculated bulk modulus (GPa), shear modulus (GPa), Young’s modulus (GPa), B/G, Poisson’s ratio and Vickers hardness (GPa) of Al_3_TM compounds.

Species	B_V_	B_R_	B	G_V_	G_R_	G	E	σ	B/G	Hv
Al_3_Sc	82.37	81.91	82.14	67.09	63.88	65.49	155.21	0.185	1.25	14.72
			84.00 [13]							
	92.1 [25]	91.8 [25]	91.9 [25]	74.1 [25]	71.7 [25]	72.9 [25]	173.1 [25]	0.19 [25]	1.26 [25]	
Al_3_Ti	105.25	105.14	105.19	91.47	84.71	88.09	206.60	0.173	1.19	19.32
			107.00 [13]							
	102.4 [25]	102.2 [25]	102.3 [25]	81.8 [25]	81.7 [25]	81.8 [25]	193.8 [25]	0.18 [25]	1.25 [25]	
Al_3_V	119.40	119.35	119.37	101.31	97.05	99.18	233.01	0.175	1.20	20.70
			118.00 [13]							
			118.32 [21]			97.22 [21]	228.95 [21]	0.178 [21]	1.217 [21]	
Al_3_Y	73.98	73.55	73.76	54.76	50.91	52.83	127.95	0.211	1.40	10.78
Al_3_Zr	101.91	101.61	101.76	87.87	80.58	84.23	198.04	0.176	1.21	18.45
	100.8 [25]	100.6 [25]	100.7 [25]	84.6 [25]	83.3 [25]	84.0 [25]	197.2 [25]	0.17 [25]	1.20 [25]	
Al_3_Nb	125.06	124.89	124.98	106.88	103.25	105.06	246.20	0.172	1.19	21.85
			124.45 [21]			103.71 [21]	243.50 [21]	0.174 [21]	1.200 [21]	
Al_3_La	60.09	56.78	58.43	25.94	18.88	22.41	59.62	0.330	2.61	1.02
Al_3_Hf	108.05	107.96	108.01	90.61	83.30	86.95	205.67	0.183	1.24	18.15
	106.5 [25]	106.0 [25]	106.3 [25]	77.8 [25]	77.4 [25]	77.6 [25]	187.2 [25]	0.21 [25]	1.37 [25]	
			107.60 [33]			86.60 [33]	204.80 [33]	0.180 [33]	1.24 [33]	
Al_3_Ta	130.22	130.18	130.21	109.53	106.85	108.19	254.17	0.175	1.20	21.94

**Table 4:** The calculated universal anisotropic index (A^U^), percent anisotropy (A_B_ and A_G_) and shear anisotropic factors (A_1_, A_2_ and A_3_) of Al_3_TM compounds.

Species	A^U^	A_B_	A_G_	A_1_	A_2_	A_3_
Al_3_Sc	0.257	0.282	2.448	1.235	1.235	1.904
Al_3_Ti	0.400	0.053	3.838	1.186	1.186	2.351
Al_3_V	0.220	0.019	2.149	1.009	1.009	1.892
Al_3_Y	0.385	0.294	3.648	1.125	1.125	2.297
Al_3_Zr	0.455	0.148	4.328	1.193	1.193	2.489
Al_3_Nb	0.177	0.067	1.727	0.994	0.994	1.763
Al_3_La	1.927	2.839	15.745	0.549	0.549	2.603
Al_3_Hf	0.440	0.043	4.203	1.185	1.185	2.457
Al_3_Ta	0.126	0.015	1.238	1.005	1.005	1.625

**Table 5:** The calculated uniaxial elastic moduli (GPa) in different directions of Al_3_TM compounds.

Species	B				G				E			
	[100]	[010]	[001]	$\left[1\bar{1}0\right]$	[100]	[010]	[001]	$\left[1\bar{1}0\right]$	[100]	[010]	[001]	$\left[1\bar{1}0\right]$
Al_3_Sc	266.74	266.74	212.26	266.74	78.78	78.78	67.20	57.33	130.84	130.84	128.50	189.79
Al_3_Ti	325.45	325.45	297.08	325.45	108.13	108.13	94.46	68.53	151.81	151.81	201.28	255.45
Al_3_V	364.46	364.46	345.91	364.46	112.85	112.85	99.37	81.44	188.96	188.96	245.33	279.07
Al_3_Y	240.26	240.26	189.66	240.26	64.17	64.17	51.60	43.06	102.58	102.58	112.72	168.70
Al_3_Zr	321.50	321.50	276.17	321.50	104.73	104.73	90.00	64.54	143.97	143.97	188.37	251.67
Al_3_Nb	387.46	387.46	351.47	387.46	117.56	117.56	104.57	88.11	206.57	206.57	255.60	292.41
Al_3_La	253.02	253.02	103.00	253.02	20.84	20.84	12.67	16.21	64.20	64.20	50.86	114.52
Al_3_Hf	333.26	333.26	306.61	333.26	107.66	107.66	92.91	66.74	148.61	148.61	201.15	257.63
Al_3_Ta	393.05	393.05	375.16	393.05	116.16	116.16	104.96	90.85	212.90	212.90	257.95	285.91

## Data Availability

The data that support the findings of this study are available from the corresponding author upon reasonable request.
